# GATA2 Inhibition Sensitizes Acute Myeloid Leukemia Cells to Chemotherapy

**DOI:** 10.1371/journal.pone.0170630

**Published:** 2017-01-23

**Authors:** Li Yang, Hanxiao Sun, Yanan Cao, Binbin Xuan, Yingchao Fan, Huiming Sheng, Wenfang Zhuang

**Affiliations:** 1 Department of Hematology, Tongren Hospital, Shanghai Jiao Tong University School of Medicine, Shanghai, China; 2 Department of Laboratory Medicine, Tongren Hospital, Shanghai Jiao Tong University School of Medicine, Shanghai, China; University of Navarra, SPAIN

## Abstract

Drug resistance constitutes one of the main obstacles for clinical recovery of acute myeloid leukemia (AML) patients. Therefore, the treatment of AML requires new strategies, such as adding a third drug. To address whether GATA2 could act as a regulator of chemotherapy resistance in human leukemia cells, we observed KG1a cells and clinical patients’ AML cells with a classic drug (Cerubidine) and Gefitinib. After utilizing chemotherapy, the expression of GATA2 and its target genes (EVI, SCL and WT1) in surviving AML cells and KG1a cells were significantly enhanced to double and quadrupled compared to its original level respectively. Furthermore, with continuous chemotherapeutics, AML cells with GATA2 knockdown or treated with GATA2 inhibitor (K1747) almost eliminated with dramatically reduced expression of WT1, SCL, EVI, and significantly increased apoptotic population. Therefore, we propose that reducing GATA2 expression or inhibition of its transcription activity can relieve the drug resistance of acute myeloid leukemia cells and it would be helpful for eliminating the leukemia cells in patients.

## Introduction

Acute myeloid leukemia (AML) is a clinically and biologically heterogeneous malignancy that is primarily treated with chemotherapy, targeted therapy, immune regulation therapy and bone marrow transplant [1, 2]. Considerable progresses have been made in the treatment of AML with application of combined chemotherapies during recent years, which manifested as improved complete remission and long-term survival rates. However, problems such as the resistance to chemotherapies and serious adverse reaction during the process of chemotherapies remain to be tackled in AML treatment[2], in which major cause of treatment failure is resistance to cytarabine (ara-C) and anthracycline [e.g., daunorubicin (DNR)]-based chemotherapy[3]. Thus, novel strategies such small molecular medicines and addition of a third drug are needed to overcome chemoresistance and improve the overall survival of AML patients[4].

GATA2 is a transcription factor crucial for hematopoietic differentiation and lymphatic formation. More specifically, GATA2 plays an essential role in maintaining the proliferation and survival of early hematopoietic cells, as well as preferential differentiation to erythroid or megakaryocytic lineages [5, 6]. Also, the expression of GATA2 is significantly higher in AML compared to normal bone marrow, and recently Maaike Luesink and his colleagues found that high GATA2 expression was a poor prognostic marker in acute myeloid leukemia [7, 8] as well as other transcription factors EVI [9, 10],WT1 [11, 12]. As for GATA2 mutations, in sporadic AML, the majority of acquired GATA2 variations were located within codon 362 in the C-terminal zinc-finger domain, which suggested that this location might be a mutation hot spot and zinc-finger domain relates to the transcriptional activity, promote the downstream genes’ expression[13, 14]. Both loss and gain of function experiments demonstrate that GATA-2 level regulates adult HSC quiescence and modulates HSC apoptosis[15], which could affect the responses to chemotherapy and contribute to poor prognosis in AML.

Our study found that GATA2 expression was associated with the resistance of AML cells to chemotherapeutic medicines. With several repeated chemotherapies, the AML cells with low GATA2 expression were almost killed, which presented a promising potential for clinical treatment. Our founding suggests that suppression of GATA2 expression or its transcriptional activity inhibition could be cooperated with the chemotherapy medicines in the treatment of AML patients.

## Materials and Methods

### Cell lines and preparation of primary AML cells

KG1a cells (human acute myeloid leukemia cell line) were cultured in RIPM1640 plus 10% FBS, and were collected at day 0, 3, 5, 7 and 9, with or without different treatments. Then cell pellets were washed twice with PBS and RIPA lysis buffer was added to get the cell lysates. Eight consecutive and unselected AML patients were investigated and the peripheral blood blast counts of these patients were more than 7×10^9^/L. Primary AML cells were isolated by density gradient separation alone (Lymphoprep, Axis-Shield, Oslo, Norway) and contained at least 95% leukemic blasts. AML cells were stored in liquid nitrogen until used in experiments[16]. The study protocol was approved by the institutional review board of the Tongren Hospital, Shanghai Jiao-tong University School of Medicine. Before the study, informed consent was obtained from each participant.

### RNA extraction

RNA from KG1a and primary AML cells was extracted using the TRIzol^®^ Reagent (Invitrogen) according to the manufacturer’s instruction. To avoid DNA contamination, RNase-free DNase I was applied. DNA/Protein Analyzer (NanoDrop, Invitrogen) assessed the RNA concentration and quantity.

### Quantitative real-time PCR

Reverse transcript was performed in a 20 μl reaction system with a total of 1 μg of RNA with M-MLV Reverse Transcriptase (TOYOBO, JAPAN). Quantitative RT-PCR was performed using ABI PCR Thermal Cycler Dice Detection System and SYBR green dye (TOYOBO, JAPAN) according to the manufacturer’s recommended protocol. The PCR primer sequences are listed in Table 1.

**Table 1:** Primers sequences used in the study.

Gene	Primers sequences
GATA2-Forward	CTACTACGACGGGGATGTTGG
GATA2-Reverse	GAGTCATGCGGATTCGGTGAG
EVI-Forward	ATGGCGTACAGTCAAGGAGG
EVI-Reverse	TGCGGATTCTATGAGGCTTCA
SCL-Forward	CCTGGCATTGACCCATAGCC
SCL-Reverse	CTCTTGGTGAAGCCTTGCATA
WT1-Forward	CAGTTCCCCAACCACTCATT
WT1-Reverse	AAGCTGGGATGTCATTTGGT
GAPDH-Forward	ACAACTTTGGTATCGTGGAAGG
GAPDH-Reverse	GCCATCACGCCACAGTTTC

### Generate stable cell lines with GATA2 knockdown

The online software (http://rnaidesigner.lifetechnologies.com/rnaiexpress/setOption.do?designOption=shrna&pid=-1447534201472129460) was used to design shRNAs of GATA2. The shRNA sequences were listed as follows: 132 GGTGGACGTCTTCTTCAATCA; 654GGAGAGCATGAAGATGGAAAG; 1187 GGAACCGGAAGATGTCCAACA. shRNAs were selected by realtime PCR and western blot, then lentivirus particle was packaged and secreted by 293T cells transfected with the shRNA, VSVG and delta8.9 package plasmids.

KG1a cells were incubated with the lentivirus mixture for 72 h, in the presence of polybrene (Sigma) at a final concentration of 8 μg/ml. Then cells were detached by trypsin and sorted with green fluorescence to get the stable knockdown cell lines. Constructed stable cell lines were amplified and stored in liquid nitrogen for future use.

### Cell viability

Cells were seeded in 96-well plates and treated with drugs (Erltinib,5μM; Geftinib,10μM; Cerubidine,1μM; Adriamycine,5μM; Idamycin,5μM; Ara-C,1μg/ml) once every day for 11 days. Cell viability was measured by the CCK-8 assay (CCK-8, Dojindo Laboratories, Kumamoto, Japan) according to the manufacturer’s instruction. All experiments were independently repeated at least 3 times. The survival rate was evaluated by cell counting kit-8 (CCK-8; Dojindo Molecular Technologies Inc., Gaithersburg, MD, USA). Cells were seeded in 48-well plates with RIPM1640 plus 10% FBS. The IC50 value was calculated using Statistical Package of the Social Sciences (SPSS) software version 12 (SPSS Inc., Chicago, IL, USA).

### Flow cytometry with annexin V-FITC and PI staining

The cells were divided into two groups: scramble (Scr) KG1a cells and KG1a cells stably knocking down of hGATA2. Each group was treated with DMSO, Geftinib (10μM) or Cerubidine(1μM). The KG1a cells were collected at day 0,3,5,7,9, after washed twice with PBS, and then stained with propidium iodide and annexin V. Apoptotic analyses were performed by flow cytometer (FCM) as described above using an FACS Calibur system. Apoptotic cells were analyzed by quadrant statistics of the propidium iodide negative and annexin V-positive cells.

### Western blotting

The whole cell lysates were extracted in 1×SDS, equally loaded to 8% to 12% SDS–polyacrylamide gel, electrophoresed, and transferred to nitrocellulose membrane (Bio-Rad) and stained with 0.4% Ponceau S red to ensure equal protein loading. After blocking with 5% nonfat milk in PBS, the membranes were incubated with antibodies. GATA2 antibody was purchased from Abcam (ab22849, Abcam, MA, USA). GAPDH antibody was purchased from Santa Cruz (SC-365062, Santa Cruz, CA, USA). β-actin was used to measure the amount of protein loaded of each sample. The signals were detected with a chemiluminescence HRP kit (Cell Signaling Technology) according to manufacturer’s instructions.

### Statistical analysis

Data are presented as the mean ± SD. Statistical analysis was performed with the Statistical Package for the Social Sciences (SPSS) software version 12 for Windows (SPSS Inc., Chicago, IL, USA). Student t-tests were used to determine the statistical significance of the differences between the experimental groups. *P*-value of <0.05 was considered significant.

## Results

### KG1a cells survived from sequential chemotherapies

**Fig 1:**
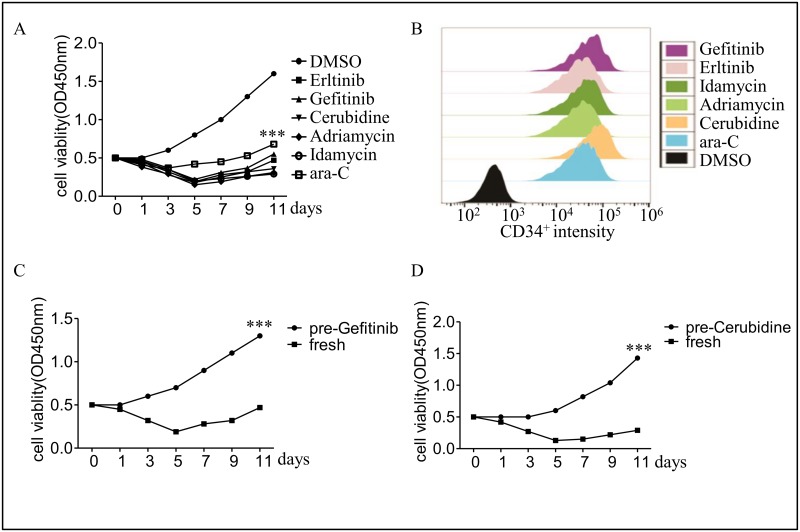
KG1a cells survived from sequential chemotherapy. (A) Cell viability was measured using the Cell Counting Kit-8, the work solution concentration of these six medicines listed as follows: Erltinib, 5 μM; Geftinib, 10 μM; Cerubidine, 1 μM; Adriamycine, 5 μM; Idamycin, 5 μM; Ara-C, 1 μg/ml. (B) The survival KG1a cells displayed higher intensity of CD34 after chemotherapy. (C and D) The survival cells from chemotherapy displayed stronger resistance to Geftinib or Cerubidine. All experiments were independently repeated at least 3 times. ***P <0.001.

KG1a cells derived from AML patients that be used as an *in vitro* model to study the mechanism of AML cells tolerance towards chemo-treatment. In order to determine the exact dosages of chemotherapy drugs used in KG1a cells, half maximal inhibitory concentration (IC50) was performed. According to our study, the IC50s of Erltinib, Geftinib, Cerubidine, Adriamycine, Idamycin and Ara-C in KG1a cells were 4.93μM, 8.92μM, 1.27μM, 4.51μM and 1.27μg/ml (S1 Fig). As shown in Fig 1A, six chemotherapy drugs: Gefitinib, Erlotinib, Cerubidine, Adriamycine, Idamycin and Ara-C were applied to treat KG1a cells respectively for 11 days. The mortality rate of these six drugs was similar except Ara-C whose ability was weaker compared with another 5 drugs. What’s more, the mortality rate of KG1a cells reached the maximum value at 80% at the fifth day. After that, the number of cells increased under continuous administration, which means that the remaining cells were capable of survival during the chemotherapies.

LSCs are commonly considered to be a kind of cells that could hardly be eliminated by chemotherapy drugs and performed as the quiescent cell in patients. KG1a cell line, which we further studied on, were proved to contain (54.167 ± 6.57)% LSCs [17, 18]. After 11 days treatment of six chemo-drugs, the intensity of CD34 fluorescence significantly increased over than 100 times in the survival KG1a cells, what’s more, cells treated with Cerubidine had the highest intensity and the ones with Gefitinib had similar fluorescence intensity (Fig 1B). Thus, these two drugs were selected for the further study.

To determine the tolerance ability of the survival cells, cells that had treated with Gefitinib and Cerubidine (pre-Gefitinib, pre-Cerubidine) were cultured with same drugs at the same concentrations for another 11 days. As shown in Fig 1C and 1D, the survival cells became much more resistant to Gefitinib and Cerubidine treatment compared with those treated first time (fresh).

### GATA2 is upregulated during the progression of clinical patients’ AML cells and KG1a treated with drugs

**Fig 2:**
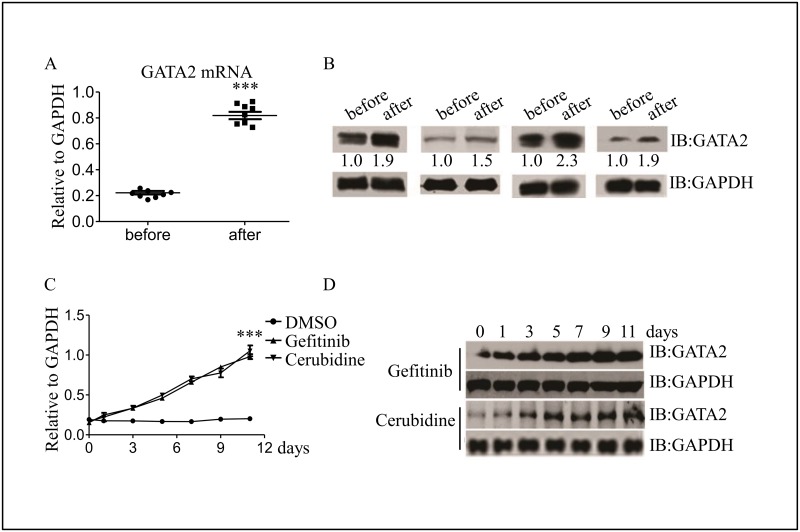
GATA2 is upregulated during the progression of clinical patients’ AML cells and KG1a cells treated with drugs. (A) GATA2 mRNA expression in peripheral primary AML cells and chemo treatment AML cells were determined by real time PCR. (B) Western blot detected the GATA2 protein level in two groups as described in Fig 2A. (C) Real time PCR identified GATA2 mRNA expression level with Geftinib(10 μM) or Cerubidine(1 μM) treatment in KG1a cells. (D) GATA2 protein expression after KG1a cells were treated by Geftinib(10 μM) or Cerubidine(1 μM) at different times. ***P <0.001.

To evaluate the potential effect of GATA2 in survival cells, we selected 8 patients who were diagnosed as untreated AML with CD34 positive or partly positive in the hospital, whose characteristics were shown in S1 Table. The peripheral primary AML cells were selected before chemotherapy and after 3 months of chemotherapy which named “before” and “after” respectively. We found that the mRNA levels of GATA2 increased about four folds in “after” samples compared with “before” samples (Fig 2A), while similar results were observated in GATA2 protein expression in peripheral AML cells (Fig 2B). All the patients were received the classic remission induction therapy, thus we intended to observe the GATA2 expression in KG1a cells treated with Gefitinib and Cerubidine respectively. As shown in Fig 2C and 2D, both the mRNA and protein expression level of GATA2 in the survival KG1a cells were kept rising trend during the second treated with Gefitinib and Cerubidine, and the statistic significant appeared one day after treatment.

### GATA2 targeting genes were unregulated during chemotherapy

**Fig 3:**
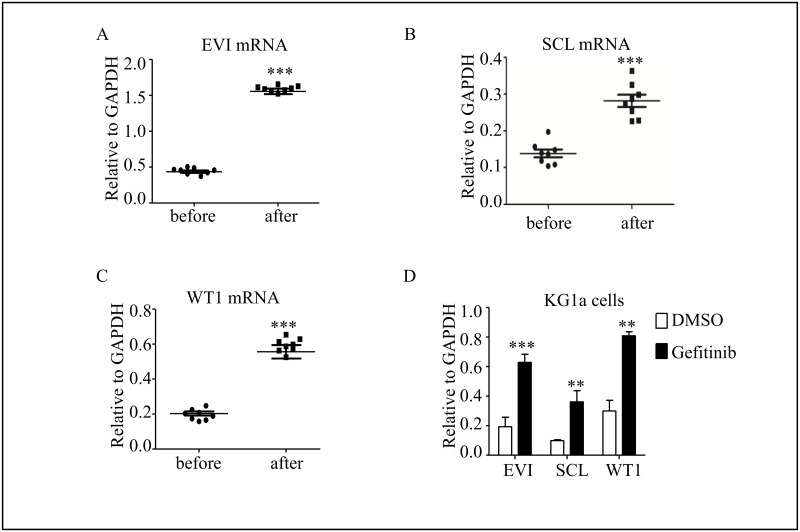
GATA2 targeting genes are up-regulated during chemo treatment. (A-C) Real time PCR identified GATA2 targeting genes: EVI, SCL and WT1 in patients’ primary AML samples. (D) Real time PCR identified the same genes in KG1a cells. Primers used to detect EVI, SCL and WT1 mRNA level were listed in materials and methods. **P <0.01, ***P <0.001.

GATA2 targeting multiple genes are associated with the onset of AML, such as EVI[9, 19], SCL[20, 21] and WT1[11], even including itself[22]. We observed that the expression levels of EVI, SCL and WT1 in peripheral primary AML cells of the same patient were surged to 3 fold after chemotherapy (Fig 3A–3C). The similar results were also detected in KG1a cells treated with Gefitinib, whose expression of these three genes were 2–4 folds higher after 11 days (Fig 3D).

### Knocking down GATA2 reduced cell viability and increased apoptosis during chemo-treatment

**Fig 4:**
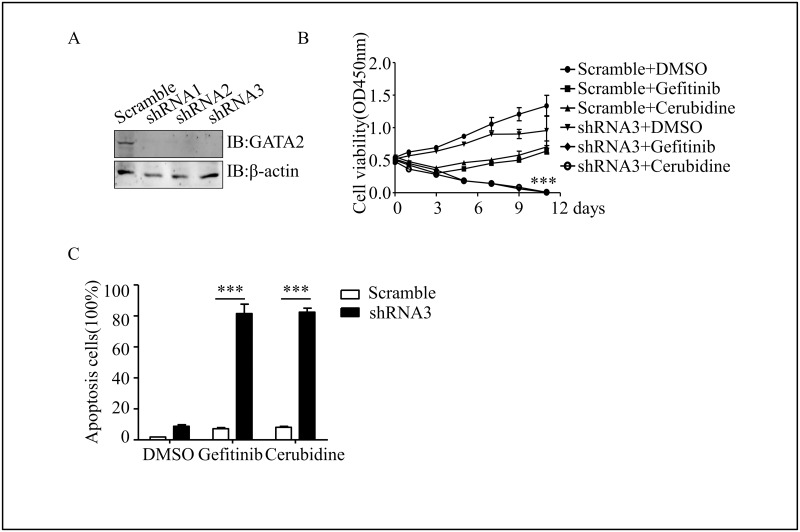
Knocking down of GATA2 in KG1a cells induced the sensitivity to chemotreatment. (A) The GATA2 knockdown efficiency was identified by western blot in KG1a cells. The sequences used to RNA interference was listed in materials and methods. (B) Cell viabilities after treatment of Gefitinib or Cerubidine between scramble and GATA2 knockdown cells were measured by CCK8 assay at different times. (C) Annexin V-FITC and PI staining determined apoptosis cells at 11 day. ***P<0.001.

Thus, is it possible that the KG1a cells could be sensitize to chemotherapy by blocking the expression of GATA2? We delivered shRNA sequence specifically targeting GATA2 into KG1a cells (Fig 4A). Furthermore, chemotherapies almost completely removed the KG1a cells with depressed GATA2 expression after 11 days of continuous chemotherapies (Fig 4B). After knocking down GATA2 in KG1a cells, Gefitinib or Cerubidine treatment showed a significant increase in the rate of apoptosis (Fig 4C). These results showed that GATA2 contributed to drug resistance in the chemo treatment of AML patients.

### GATA2 inhibitor reduced gefitinib-induced GATA2 and its targeting genes expression in clinical primary AML cells

**Fig 5:**
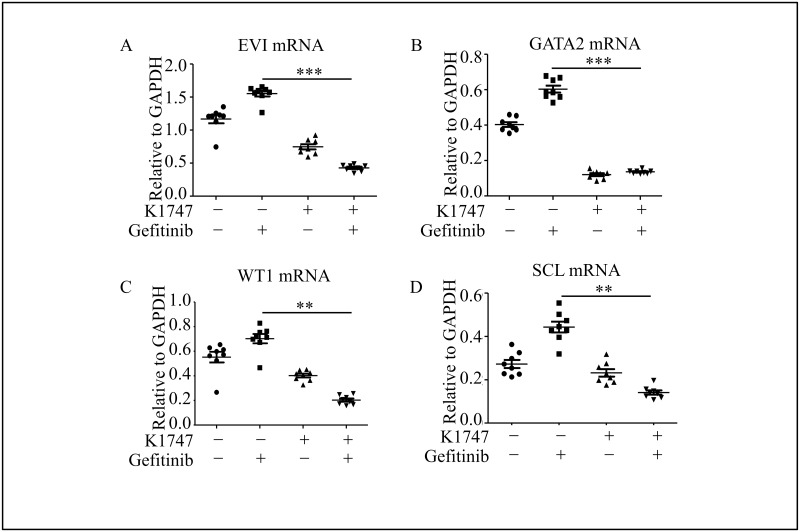
Inhibition of GATA2 in clinical primary AML cells decreased the survival rate during chemo treatment. (A-D) Combination of GATA2 inhibitor and chemotherapies (Gefitinib) dramatically reduced AML related-genes expression as described in Fig 3. Geftinib = 10 μM; K7174 (GATA2 inhibitor) = 20 μM.**P<0.01, ***P<0.001.

The expressions of GATA2, WT1, SCL and EVI were evaluated in clinical primary AML cells treated with or without GATA2 inhibitor (K1747) and Gefitinib. Results showed that EVI, GATA2, SCL and WT1 were upregulated after Gefitinib treated, while the combination of K1747 and Gefitinib dramatically reduced the expression of them (Fig 5A–5D). What’s more, we observed that the EVI, SCL and WT1 expression reduced in different extent when K1747 was added with six drugs mentioned above (S2 Fig). However, EVI and WT1 levels were the lowest in KG1a cells with K1747 and Gefitinib, compared with the ones with other drugs. Therefore, it is possible to clean up either the AML cell line or the primary AML cells by combining certain chemotherapy drugs and GATA2 inhibitor together, especially Gefitinib.

## Discussion

Recently, GATA2 is proved to be required for the survival of RAS pathway mutated non-small cell lung cancer (NSCLC) cells [23], and it has identified that GATA2 as a regulator of chemotherapy resistance and tumorigenicity in lethal prostate cancer through experimental models and clinical databases [24]. While GATA2 has long been implicated as a hematopoietic transcription factor and its dysregulated expression is associated with human immunodeficiency syndromes and vascular integrity [25], thus, the regulate effect of GATA2 observed in solid tumors may have similar effect on human leukemia cells to chemotherapy. To address whether the effect could be replicated in human leukemia cells, we oberserved KG1a cells and clinical patients’ AML cells with a classic drug (Cerubidine) and Gefitinib [26, 27].

Studies have clearly demonstrated that AML cells are propagated by a subpopulation of leukemia stem cells (LSCs) [28]. Initial reports described a specific cell surface phenotype for LSCs that allowed primitive leukemia cells to be distinguished from normal stem and progenitor cells (CD34+, CD38-, CD123+ and so on)[29]. It has been reported that LSCs are substantially more tolerated to multiple chemotherapies than bulk leukemia populations [28]. Laboratory studies have specifically examined challenge with cytarabine and daunorubicin and have demonstrated preferential survival of functionally defined LSCs [30]. Our data proved that the survival leukemia cells from chemotherapies presented much higher level of CD34+, indicating that there are a higher proportion of stem / progenitor cells in these residual population cells. Unfortunately, we failed to make this result more reliable because no other cell surface phenotype was observed. Thus, there is no solid evidence to prove the relationship between high expression of GATA2 and the progress of LSCs.

In the current study, we found that human leukemia cells that resistant to traditional chemotherapy demonstrated upregulated GATA2. This finding echoes the results reported from human hematopoietic stem and progenitor cell study showing that sustaining high GATA-2 expression confers increased quiescence and consequently limits their performance[31]. Our research confirmed GATA2 expression in the survival cells dramatically increased to 2–4 folds comparing to the control group after the second treatment, which suggested GATA2 might involve to the drug resistance. What’s more, our research suggested that the combination with suppression of GATA2 expression or transcriptional activity and chemotherapies could be a novel strategy to improve the complete remission rate and benefit long-term survival of patients, such as reducing the expression of GATA2 by GATA2 5 'mirRNA UTR, or applying the method of crisper/cas9 to knock out GATA2' UTR [32].

Apoptosis is a form of cell death that could eliminate cells without releasing inflammatory substances into the surrounding area. Previous report showed that TIM3+ HSCs with higher GATA2 displayed overproliferation and decreased apoptosis [33]. Our result confirmed that the increased apoptosis appeared in KG1a cells with knocking down GATA2. However, the fact that we did not explore the underlying mechanism is a limitation of the current study; thus, it is not clear the exact pathway that cause GATA2 gene expression surge after chemotherapy and affect apoptosis. We make a hypothesis that there is a positive feedback mechanism to regulate GATA2 expression: GATA2 expression is upregulated by targeting to itself promoter while chemotherapy promotes GATA2 transcriptional activity. There is evidence that GATA2 targets to its own promoter [34], but under what conditions, such as extracellular stress or intracellular stimuli are still elusive. Thus, the hypothesis is that during the process of chemotherapy with AML cells, GATA2 is activated and binds to its own promoter which in turn enhanced the expression of GATA2 itself. After a series of chemotherapies, a high level of activated GATA2 might confer a strong resistance to the chemotherapy, which eventually leads to failed chemotherapies.

Our study remains to be validated in clinical trials, but it offers an appealing orientation: several nodes of the GATA2 signaling pathway are amenable to existing approved clinical agents, in turn facilitating translation to clinical therapies. Furthermore, the challenge to identify the optimal combinations of chemo-medicines to suppress the proliferation and survival of AML cells remains to be solved.

## Supporting Information

S1 Fig IC50 values of the six drugs in KG1a cells. KG1a cells were treated with various doses of six drugs for 48 hours and IC50 was determined by CCK8 assay.
(TIF)

S2 Fig Combination K1747 and chemotherapeutic drugs affect EVI, SCL and WT1 mRNA expression. After KGla cells were treated with K1747 and chemotherapeutic drugs for 48 hours, EVI, SCL and WT1 mRNA expression was measured by real-time PCR. The drug concentrations were described as Fig 1.
(TIF)

S1 Table Characteristics of clinical patients. (XLSX)
