# Correction: GATA2 Inhibition Sensitizes Acute Myeloid Leukemia Cells to Chemotherapy

**DOI:** 10.1371/journal.pone.0175005

**Published:** 2017-03-28

**Authors:** Li Yang, Hanxiao Sun, Yanan Cao, Binbin Xuan, Yingchao Fan, Huiming Sheng, Wenfang Zhuang

The authors have incorrectly referenced a GATA2 Inhibitor as K1747 throughout the paper and supporting information files. The correct GATA2 inhibitor is K7174.
