# Supplementary figures and images for: GATA2 Inhibition Sensitizes Acute Myeloid Leukemia Cells to Chemotherapy
Source: PLoS One. 2017 Jan 23;12(1):e0170630. doi: 10.1371/journal.pone.0170630 (PMC5256934; doi:10.1371/journal.pone.0170630)

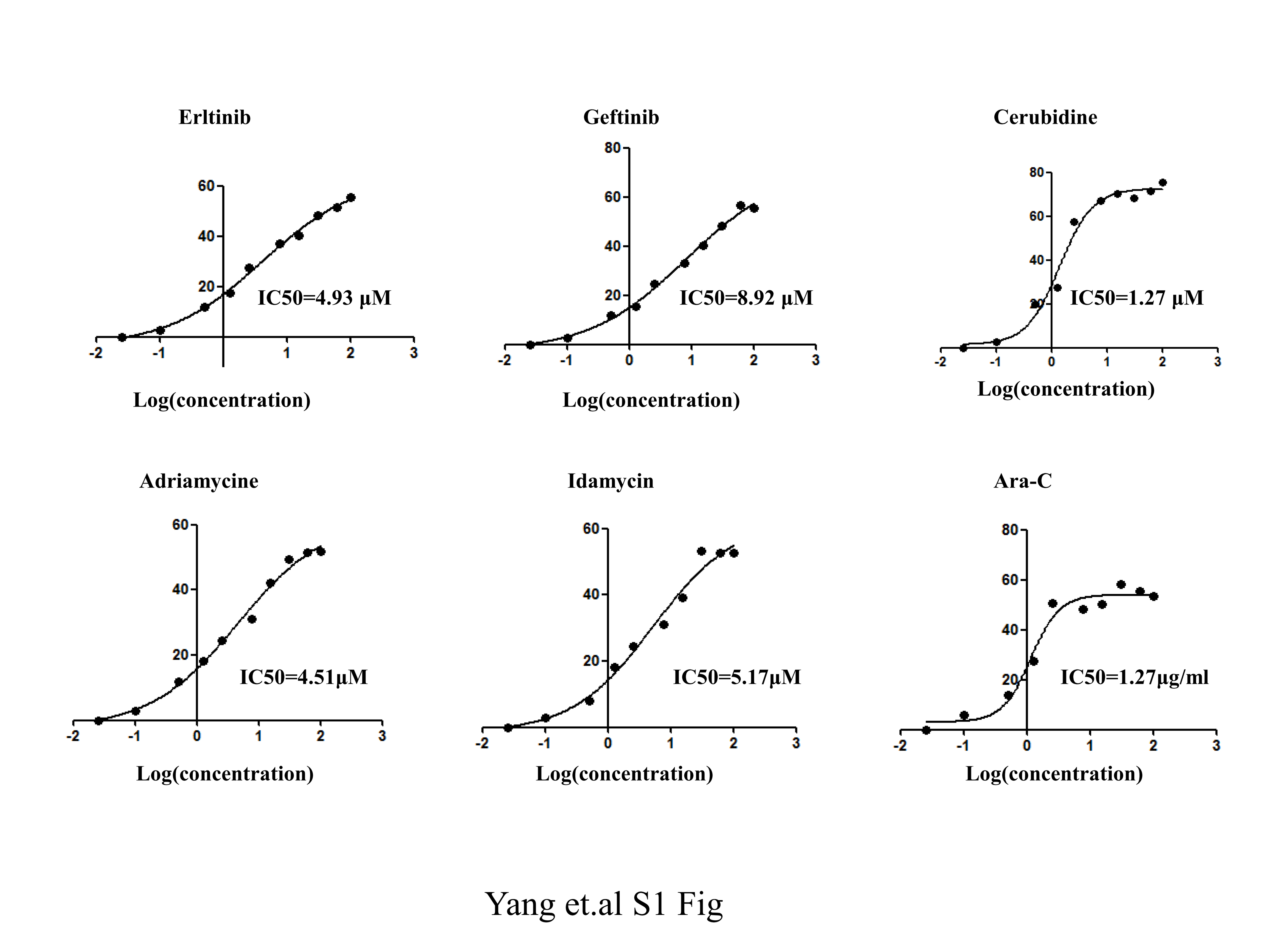

Supplement: S1 Fig — KG1a cells were treated with various doses of six drugs for 48 hours and IC50 was determined by CCK8 assay. (TIF) [file pone.0170630.s001.tif]

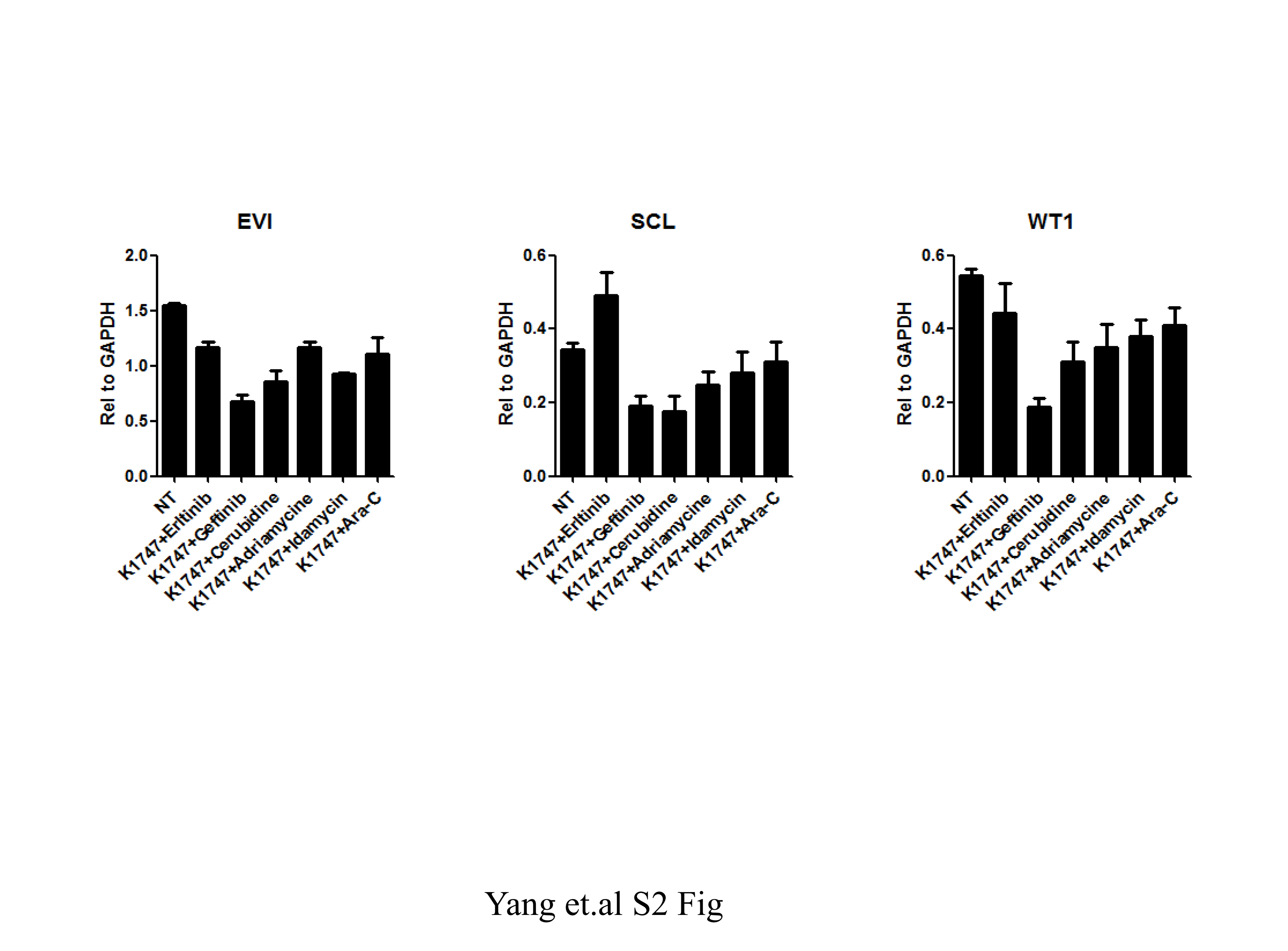

Supplement: S2 Fig — After KGla cells were treated with K1747 and chemotherapeutic drugs for 48 hours, EVI, SCL and WT1 mRNA expression was measured by real-time PCR. The drug concentrations were described as Fig 1. (TIF) [file pone.0170630.s002.tif]
